# Western Ontario Osteoarthritis of the Shoulder Index (WOOS) - a validation for use in proximal humerus fractures treated with arthroplasty

**DOI:** 10.1186/s12891-023-06578-5

**Published:** 2023-06-02

**Authors:** Yilmaz Demir, Hanna Sjöberg, Andre Stark, Björn Salomonsson

**Affiliations:** Karolinska Institutet, Stockholm, Sweden

**Keywords:** Proximal humerus fracture, Shoulder arthroplasty, Shoulder surgery, Patient reported outcome and validation

## Abstract

**Background:**

The Swedish shoulder and Arthroplasty Registry (SSAR) use the Western Ontario Osteoarthritis of the Shoulder Index (WOOS) as their shoulder-specific score in the follow-up. WOOS is not yet validated for use as the Patient Reported Outcome Measurement (PROM) for proximal humerus fractures (PHF) treated with shoulder hemiarthroplasty (SHA) in the Swedish registry. The aim of this study was to examine the validity, the reliability and the responsiveness of WOOS as a PROM for proximal humerus fractures treated with shoulder arthroplasty.

**Methods:**

Data was collected from the SSAR from the 1^st^ of January 2008 to the 31^st^ of June 2011. A total of 72 subjects were identified with at least 1 year of follow-up. Of these 43 completed all the shoulder-specific PROM together with a clinical examination, including a WOOS retest and general health scores. A group of 29 did not undergo any clinical examination, but they completed all the questionnaires not requiring a clinical examination. The validity was assessed with WOOS compared to satisfaction level, and the Spearman rank coefficient was used for the correlation between WOOS and the shoulder-specific scores (Constant-Murley Score, Oxford Shoulder Score, American Shoulder and Elbow Surgeons Standardized Shoulder Assessment Form and EQ-5D. For reliability, Intra Class Correlation (ICC) was used for the test–retest assessment and Cronbach´s alpha for the construct reliability.

**Results:**

The validity for WOOS had an excellent correlation (> 0.75) with all the shoulder-specific scores and a good correlation (> 0.6) with EQ-5D.

The reliability with the test–retest of the total WOOS score and the subgroups had an excellent correlation. Cronbach´s alpha also supports the construct of WOOS. There were no floor or ceiling effects.

**Conclusions:**

We found that WOOS is a reliable tool for evaluating patients with SHA after PHF. Based on our study, we recommend the continued use of WOOS in shoulder arthroplasty registries and observational studies.

## Background

Patient reported outcome measures (PROM) are used to assess the effect of a treatment, and they seek to give results not biased by the caregiver´s opinion. Typically, a PROM is constructed to cover expected results for function, as well as the well-being of the patient.

The Western Ontario Osteoarthritis of the Shoulder Index (WOOS) was developed as a shoulder-specific score as an outcome in the treatment of osteoarthritis of the shoulder [1, 2].

In 2004 the Swedish Shoulder Arthroplasty Registry (SSAR) began to use a Swedish translation of WOOS as the main PROM for the registry follow-up. In addition to WOOS, the registry also collects EuroQol-five dimensions (EQ-5D) and Satisfaction Level (SL).

WOOS was originally not constructed or validated to be used for patients treated with arthroplasty after proximal humerus fracture (PHF). However, as well as osteoarthritis WOOS has also proven itself to be useful in other diagnoses, for example, rotator cuff syndrome [3, 4].

A validation of a PROM in the Swedish language, and specific for proximal humerus fractures treated with arthroplasty [5] is lacking, we wanted to assess WOOS for PHF treated with arthroplasty.

The aim of this study was to examine the validity, the reliability and the responsiveness of WOOS as a PROM for proximal humerus fractures treated with shoulder arthroplasty.

## Methods

This observational study identified patients treated with hemiarthroplasty after acute fracture from the 1^st^ of January 2008 until the 31^st^ of June 2011. Those subjects with a minimum of 1 year to 5 years follow-up period were selected and invited to participate. The time to follow-up was considered sufficient as the patients had reached a stable outcome after fracture and arthroplasty surgery as found in a multicenter study from Jonson et al. [6].

### Inclusion criteria

In the SSAR, we identified 131 patients treated at either Danderyd hospital or Karolinska Hospital Solna. These hospitals were chosen as they provided the possibility for the subject to a clinical examination carried out by the same physiotherapist at Danderyd hospital.

### Exclusion criteria

**Fig. 1:**
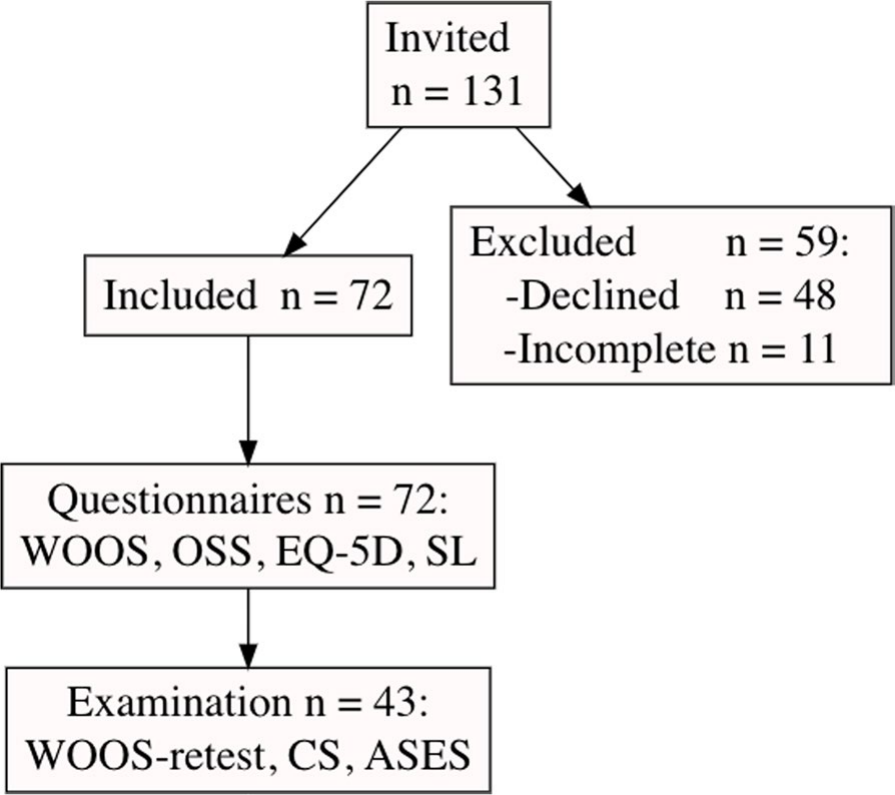
Flow-chart showing inclusion and exclusion in the study. Abbreviations: ASES – American Shoulder and Elbow Surgeons Standardized Shoulder Assessment Form, CS – Constant-Murley Score EQ-5D—generic EuroQol five dimensions health score, OSS—Oxford Shoulder Score, SL – Satisfaction level and WOOS—Western Ontario Osteoarthritis of the Shoulder Index

If the patients declined participation or if the questionnaires were incomplete, Fig. 1.

### Study population

A total of 72 subjects were included in the study. All 72 subjects completed WOOS, OSS, EQ-5D, and SL, Fig. 1. Of the included 72, a total of 43 conducted the clinical examination and the WOOS retest. A physiotherapist at Danderyd hospital did the examinations and then the subjects were handed a WOOS retest, Fig. 1.

### PROM and instruments for validation

*Western Ontario Shoulder Score* (WOOS) is a questionnaire that consists of 19 questions; 6 questions evaluate physical symptoms, 5 questions evaluate sport/activities and work, 5 questions evaluate lifestyle, and 3 questions evaluate emotions [1, 7]. It is a self-evaluating PROM questionnaire with no clinical examination.

Every question is answered using a VAS scale. 0 is the best result and 100 is the worse result. The total score is calculated where 0 is a normal healthy shoulder and 1900 is the worst possible result.

WOOS% (% of raw score) can be calculated using the formula (1900 – the score)/19 and then 0% is the worst possible result and 100% is the best possible result. We choose the WOOS% for the presentation of the results.

*Oxford Shoulder Score* (OSS) [8, 9] consists of 12 questions with 5 level answers (each question 0–4). It is a self-evaluating PROM questionnaire with no clinical examination. The total score is then presented as a number ranging from 0–48 and the lower the score the better the result.

*EuroQol five dimensions health score* (EQ-5D) assesses the general health of the patient. EQ-5D 3L consists of 5 domains with 3-level answers in each. It is a self-evaluating PROM questionnaire with no clinical examination. The EQ-5D index was calculated using the original tariff used in the UK. The 5 responses provide an index with a maximum value of 1.0. The range is from -0.53–1, and scores less than zero is considered to be a state worse than death. The SSAR also uses the EQ 5D as shoulder function may also affect the subject’s general health and quality of life [10, 11].

*Satisfaction level* (SL) was also collected, using a 5-step Likert scale (very unsatisfied to very satisfied. We dichotomized SL into two groups: the *unsatisfied group* (1 = very unsatisfied and 2 = somewhat unsatisfied) and *the satisfied group* (3 = neither satisfied nor unsatisfied, 4 = somewhat satisfied, and 5 = very satisfied). We considered SL = 3 “neither satisfied nor dissatisfied” as belonging to the satisfied group since treatment of a fracture aim to avoid unsatisfied results despite the probably negative effect following serious trauma.

*Constant-Murley Score* (CS) has a range from 0–100, The higher the score the better the shoulder function. The self-assessment/subjective part gives a maximum of 35 points while the objective measurements give a maximum score of 65 points [12–17]. CS is recommended in Europe and has been widely used for a long period [18–20]. We used the raw score without any adjustments.

*American Shoulder and Elbow Surgeons Standardized Shoulder Assessment Form* (ASES) [12–14] the possible score ranges from 0–100, the higher the score the better. It consists of 17 subjective questions and has also an objective part that includes a clinical examination. When summarizing the scores, only the subjective answers are counted. There is a ceiling effect reported with ASES, but it is < 15%. ASES is recommended in North America and has been widely used for a long period [14].

### Validity

To validate the construct of WOOS for proximal humerus fractures treated with arthroplasty, we compared WOOS with other shoulder-specific scores; OSS, ASES, and CS. We also compared WOOS with general scores such as EQ-5D 3L and SL.

### Floor and ceiling effect

To show any floor and ceiling effects, at least 15% or more of the answers should reach minimum or maximum scores [15].

### Reliability

We evaluated the total WOOS score and each WOOS domain with Cronbach’s alpha. This was done to analyze the construct reliability if the questionnaire items score adequately as a PROM for fractures operated with SHA without redundancy in the questions.

To assess the correlation and agreement between measurements a test–retest with Intra Class Correlation (ICC) for the total WOOS score was used. The first test was sent by mail for the patient to fill out a WOOS. The retest was filled out during the clinical examination. The time difference between the two tests should be sufficient to allow enough time for the subjects to forget their initial replies, while still being close enough to ensure that the symptomatology remains unchanged. We, therefore, had a minimum of 2 weeks interval between the test and the retest.

### Responsiveness

The previously reported Minimal Detectable Change (MDC) for WOOS% is about 10% and the Minimal Clinical Important Difference (MCID) is about 8% [16]. Since fracture cases do not have a PROM taken before their trauma occurs, we compared the dichotomized SL and all other scores using Spearman correlation, presented as a table.

We present responsiveness for WOOS% as a threshold for the lowest Patient Accepted Symptom State (PASS) for a patient [17–20]. We choose SL = 3 “neither satisfied nor dissatisfied” as the threshold for PHF arthroplasty to describe a value for PASS.

### Statistics

We tested all PROM for normality with Shapiro-Wilks and except for ASES, they were not normally distributed. Therefore, we used Spearman´s rank correlation for comparison of the PROM. We considered a Spearman correlation coefficient (R) > 0.75 as excellent [21].

For reliability and internal consistency, Cronbach's alpha was calculated as a measure of internal consistency to examine the relationship between a set of items representing a group [22] In a psychometric test a score > 0.9 might indicate that there is a redundancy of questions [23, 24].

We analyzed the WOOS test–retest with ICC and considered a score > 0.90 to be excellent reliability.

We dichotomized patient satisfaction SL to the groups unsatisfied and satisfied. For responsiveness, each score was compared to the dichotomized SL and presented in a table with mean and 95% confidence interval (CI 95%), and PASS was presented to match WOOS% with SL = 3 “neither satisfied nor dissatisfied”.

We used SPSS ver. 28 IBM co.

## Results

The patients where all treated with SHA for PHF and examined 1–5 years after the surgery, Table 1.

**Table 1:** Characteristics of the study group

	**Questionnaires**	**Examination**	**Total**
	**group**	**group**	
**Sex**			
Male	10	11	21
Female	19	32	51
**Age**			
In years	74 (36–88)	69 (36–90)	72 (36–90)
Median and range			
**Side**			
non-dominant	12	19	31 (44%)
dominant	15	24	39 (56%)

### Validity

WOOS% had a Spearman correlation coefficient R > 0.75 compared to all the shoulder-specific scores, and we considered this to be an excellent correlation. With OSS (since lower values are better), Spearman’s coefficient comparing WOOS% is negative. EQ-5D had *R* = 0.68 and this was considered a good correlation, Table 2.

**Table 2:** Correlation between WOOS% and the other scores with a Spearman´s rank correlation

	**OSS**	**CS**	**ASES**	**EQ-5D**
	*n* = 72	*n* = 72	*n* = 43	*n* = 70
**WOOS%**	-0.830	0.780	0.812	0.679
*n* = 72	-0.830	0.780	0.812	0.679

*Abbreviations*: *ASES* American Shoulder and Elbow Surgeons Standardized Shoulder Assessment Form, *CS* Constant-Murley Score, *EQ-5D* generic EuroQol five dimensions health score, *OSS* Oxford Shoulder Score, *WOOS* Western Ontario Osteoarthritis of the Shoulder Index

### Floor and ceiling effect

None of the subjects had a floor or ceiling effect in the shoulder-specific scores, but for EQ-5D 15.7% reached a ceiling effect.

### Reliability - Reliability with internal consistency

The internal consistency had a good to excellent correlation with Cronbach alfa for WOOS total was 0.969 and within the four domains of WOOS: Physical symptoms 0.901, sport/activities and work 0.894, lifestyle 0.907, and emotions 0.965.

### Reliability with test–retest

**Fig. 2:**
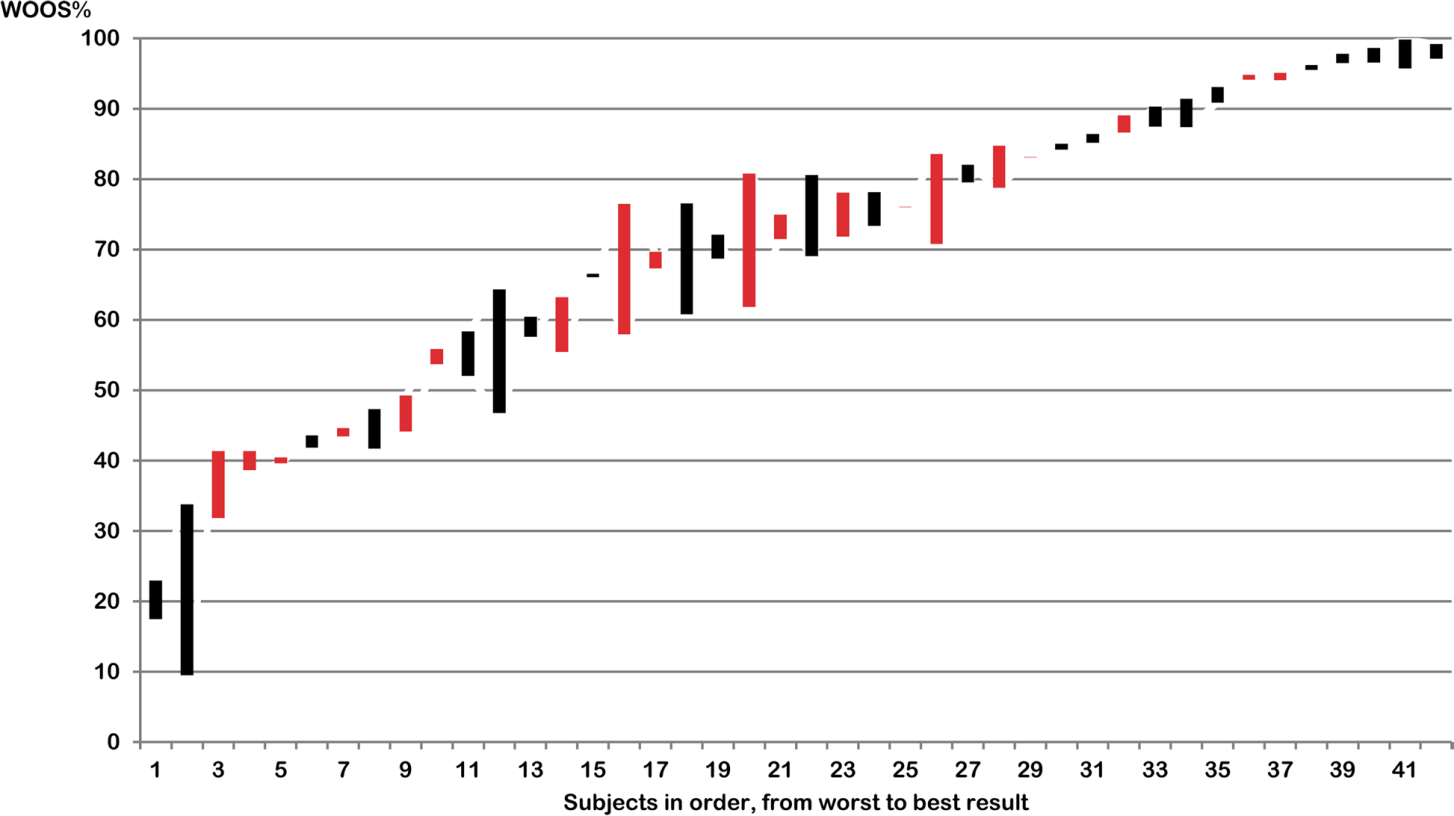
The test–retest with total WOOS%. The x-axis represents each subject individually, the height of the bars represents the difference in the test–retest, and the height represents the difference between the tests. The red color represents a positive difference, and the black color represents a negative difference when subtracting the result of the test from the re-test. Abbreviations: WOOS—Western Ontario Osteoarthritis of the Shoulder Index

ICC for test–retest of total WOOS% score showed excellent reliability, 0.970 (CI_95_ 0.944—0.984). The test–retest scores are illustrated in Fig. 2.

### Responsiveness

WOOS%, CS, OSS, ASES, and EQ-5D with Satisfaction level as total scores are compared with a dichotomized SL. The result of all the questionnaires showed a good correlation with the level of satisfaction, Table 3.

**Table 3:** Table of the scores for Satisfaction level dichotomized

**The mean outcome (with CI 95%) of the different scores**			
	**Satisfied *n* = 49**	**Unsatisfied *n* = 21**	**Total**
**WOOS%** 0–100	78 (74–83)	48 (37–59)	69 (63–74)
**OSS** 0–48	10 (8–13)	25 (21–29)	15 (12–17)
**CS** 0–100	57 (50–63)	33 (26–41)	48 (42–54)
**ASES** 0–100	74 (66–81)	52 (41–63)	66 (59–72)
**EQ-5D** -0.53–1	0.77 (0.71–0.83)	0.52 (0.36–0.67)	0.68 (0.62–0.75)

WOOS%, CS, OSS, and EQ-5D: higher values are better. The values of OSS: lower values are better

*Abbreviations*: *ASES* American Shoulder and Elbow Surgeons Standardized Shoulder Assessment Form, *CS* Constant-Murley Score, *EQ-5D* generic EuroQol five dimensions health score, *OSS* Oxford Shoulder Score, *WOOS* Western Ontario Osteoarthritis of the Shoulder Index

### PASS, Patient Accepted Symptom State

With an anchor set at SL = 3 “neither satisfied nor dissatisfied” the mean PASS with CI_95_ WOOS% is 72.1 (62.1, 81.5), with at least 1-year follow-up.

## Discussion

Our findings show good validity and reliability for the Swedish version of WOOS in the follow-up of PHF treated with arthroplasty.

The strongest correlation between the shoulder-specific scores was found for WOOS and OSS. The probable explanation could be that WOOS, and OSS have similar content and intention.

A lower, but still high correlation was seen with EQ-5D and CS. As the ASES and CS scores include objective measurements, their construct differs from WOOS and OSS. Their objective measurements could be seen as a bias in multicenter studies since they would be assessed by different individuals. There is also a risk of loss to follow-up because of the need for a clinical examination. Therefore, using these in a national registry may also be impractical. The correlation with EQ-5D was expected to be lower than the shoulder the specific PROM as it is not a shoulder-specific score. However, our results show that decreased shoulder function and pain also affect the subject’s general health.

Since our findings show a Cronbach’s alpha close to l in each domain and the ICC with test–retest [25] was also close to 1, it indicates high reliability for the total WOOS score. Our results could also exclude the time elapsed between the test and the retest as a confounder.

PROM are important to give patients a voice and caregivers a tool to evaluate the disease or treatment in the registries [26–28].

Our study of PHF treated with arthroplasty lacks adequate data on the PROM of shoulder function before the trauma and fracture occurred. In general, a fracture is expected to reduce function even after successful treatment. Therefore, we cannot calculate a verified treatment effect or provide any measure of treatment improvement. In this study, we calculated the Patient Acceptable Symptom State (PASS) to be a WOOS% of 72% at a minimum of one year after surgery, as a measure of the patient's perceived acceptable outcome. However, shoulder scores have been assessed for responsiveness, the minimum clinically important difference (MCID), as well as the effect size (ES) and substantial clinical benefit (SCB) in shoulder arthroplasty for elective surgery, with results supporting their use.

In a Danish study of WOOS in anatomical total shoulder arthroplasty for osteoarthritis, the MCID was found to be 12.3% [29]. Another study of mixed diagnoses and elective arthroplasties, comparing WOOS and two other PROMs (ASES and CS), including SCB in the analysis, found that all three measures demonstrated good to excellent responsiveness and optimal sensitivity to change [30]. Other studies that did not specifically include WOOS, but several other common shoulder outcome metrics, have also found adequate results when assessing MCID, SCB, and ES for shoulder arthroplasty treatment, although not for PHF [31–33].

Due to the lack of comparison between PHF and arthroplasty treatment for WOOS, we have to assume that there may be support for our findings in studies of outcome metrics in general regarding shoulder arthroplasty. However, further studies of PROMs after arthroplasty for PHF are needed.

### Strengths

One strength of our study is the availability of a sufficient number of patient-reported outcome measure (PROM) answers, allowing us to validate the WOOS for PHF treated with arthroplasty. Additionally, we were able to analyze the correlation between WOOS and three shoulder-specific scores as well as two general outcomes.

We consider the patients included in this study to be representative of the treatment group, and those who participated reflected a range of outcomes, spanning from low to high. The average age of the participants was 70 years, and there were slightly more females than males in the stud [34–36].

### Limitations

There was a selection bias as all the patients came from the same urban area. However, in a relatively homogeneous country like Sweden, patient characteristics did not differ greatly. Additionally, our primary focus was on assessing the PROMs rather than the treatment itself or the patients. Therefore, this should not be considered a hindrance to the generalizability of the study.

While there was a large number of patients who declined to participate in the study, the number of PROM answers obtained was sufficient to perform the analysis.

As is common in fracture treatment, we were unable to obtain a verified pre-fracture PROM, and the study lacks the ability to assess minimum clinically important difference (MCID), substantial clinical benefit (SCB), and effect size (ES).

## Conclusion

WOOS is a reliable tool for evaluating patients after proximal humerus fractures treated with shoulder arthroplasty, and may help to a understanding of the outcome which a patient perceives after treatment with arthroplasty after PHF. Based on our study, we recommend the continued use of WOOS in shoulder arthroplasty registries and observational studies.

## Data Availability

The datasets used and/or analyzed during the current study is available from the corresponding author.

## Abbreviations

ASES: American Shoulder and Elbow Surgeons Standardized Shoulder Assessment Form
CI 95%: Confidence Interval set as 95%
CS: Constant-Murley Score
ES: The Effect Size
EQ-5D: Generic EuroQol five dimensions health score
MCID: Minimum clinically important difference
OSS: Oxford Shoulder Score
PHF: Proximal humerus fracture
PASS: The Patient Acceptable Symptom State
PROM: Patient Reported Outcome Measurements
R: Spearman rank Correlation
SCB: Substantial Clinical Benefit
SL: Satisfaction Level
SSAR: Swedish Shoulder Arthroplasty Registry
WOOS: Western Ontario Osteoarthritis of the Shoulder Index
